# In the loop: Practices of self-monitoring from accounts by trial participants

**DOI:** 10.1177/1363459315611939

**Published:** 2015-10-13

**Authors:** Rebecca Lynch, Simon Cohn

**Affiliations:** University of Cambridge, UK; London School of Hygiene & Tropical Medicine, UK

**Keywords:** feedback, monitoring, physical activity, public health intervention, self-monitoring

## Abstract

Self-monitoring, by which individuals record and appraise ongoing information about the status of their body in order to improve their health, has been a key element in the personal management of conditions such as diabetes, but it is now also increasingly used in relation to health-associated behaviours. The introduction of self-monitoring as an intervention to change behaviour is intended to provide feedback that can be used by individuals to both assess their status and provide ongoing support towards a goal that may be formally set or remains implicit. However, little attention has been paid to how individuals actually engage in the process or act upon the information they receive. This article addresses this by exploring how participants in a particular trial (‘Get Moving’) experienced the process and nature of feedback. Although the trial aimed to compare the potential efficacy of three different monitoring activities designed to encourage greater physical activity, participants did not present distinctly different accounts of each intervention and the specifics of the feedback provided. Instead, their accounts took the form of much more extended and personal narratives that included other people and features of the environment. We draw on these broader descriptions to problematise the notion of self-monitoring and conclude that self-monitoring is neither solely about ‘self’ nor is it exclusively about ‘monitoring’. We suggest that a more expansive social and material understanding of feedback can give insight into the ways information is made active and meaningful for individuals in their everyday contexts.

## Introduction

It is now well established that lack of physical activity is a major public health problem, causally associated with coronary heart disease, diabetes, osteoporosis and some cancers (Ekelund et al., 2015; Nelson et al., 2002). The World Health Organization (WHO) has accordingly recognised physical inactivity as the fourth most important risk factor for noncommunicable disease, after tobacco use, hypertension and high blood glucose (WHO, 2009). However, Bell and Bauman have suggested that physical inactivity remains a ‘Cinderella’ risk, since it currently does not receive commensurate levels of policy attention or public health resource (Bell and Bauman, 2011). One of the problems is that becoming more physically active is inherently more complex than simply ‘choosing’ to meet daily recommendations (Jallinoja et al., 2010). Given this, a current public health challenge is to design interventions that promote sustained increases in levels of physical activity for an entire population.

Because such interventions need to be reproducible, scalable and affordable, those which might not depend on regular face-to-face human contact have been a key focus of recent attention. One proposal has been to explore the potential role of self-monitoring; examples include the use of pedometers and other specially designed electronic devices, smart phone apps (including geographic information system (GIS) mapping) and dedicated diary-keeping. Generally framed within psychology as the introduction of an individualised learning experience that can promote certain behaviours, feedback is said to facilitate ‘rational’ reactions to a stimuli in order to support the experience of ‘self-efficacy’, rather than rely on automatic conditioning (Kazdin, 2013). The approach thereby rests on the argument that self-monitoring can result in sustained, deliberative attempts to match behaviour to either formally set goals or more implicit ones, as long as there are positive attributions and expectations to support the attempts (Boutelle et al., 1999; Kanfer, 1991; Snyder, 1974). It consequently relies on the conceptualisation of a cybernetic-style feedback loop, by which the reflection or evaluation of a current or recent status against a reference or goal (Cameron and Levanthal, 2003) is intended to lead to a reduction, over time, between the two (Carver and Scheier, 1982; Cone, 1999).

However, while the self-management of a wide range of existing conditions is now a commonplace strategy (The Health Foundation, 2011), using self-monitoring to change unhealthy behaviours of people who are not currently ill is arguably a very different process. In particular, this shifts the imperative from trying to attain a previous state or maintain the status quo to working towards a future that might be experienced as imaginary. Thus, although regular self-monitoring has been reported by some to be successful, its current efficacy as a general form of behaviour intervention is far from clear. For example, while some weight loss studies generally report success (Baker and Kirschenbaum, 1998; Boutelle et al., 1999; Boutelle and Kirschenbaum, 1998), its ability to increase levels of physical activity has been less clear; some report a positive effect (e.g. Aittasalo et al., 2005; Speck and Looney, 2001), while others report no significant increase (e.g. Gleeson-Kreig, 2006).

Although many of these studies report that monitoring appears to be popular with many users (Kinmonth et al., 2008; Knowler et al., 2002; Lewis et al., 2008), critics of the rise and potential utilisation of self-monitoring for health interventions have argued that this merely represents a new wave of medicalisation, in which, assisted by rapidly developing forms of technology, more and more aspects of everyday life are subject to a medical gaze. In addition, by placing the burden of surveillance on individuals themselves, including leaving people to report their own successes and failures, opponents argue it is merely an extension of Foucauldian disciplinary biopower and hence a surreptitious form of governmentality (Lupton, 1997; May et al., 2006).

Our concern with self-monitoring as a mode of public health intervention is somewhat different. Although the psychological theories underlying self-monitoring interventions have, in recent years, been augmented to incorporate greater complexity, we nevertheless argue that the general approach continues to rest on three implicit assumptions: first, that people tend to be coherent and reflexive about their behaviour, which is taken to be an outcome of rational choice; second, that what is termed self-monitoring is, by definition, an individual process and that feedback occurs in a relatively closed, egocentric, loop; and third, that the specific material techniques used to undertake monitoring are not formative in the process of self-recording or evaluation, but rather are merely pragmatic aspects that may or may not have a confounding influence. Ultimately, these assumptions stem from the way in which self-monitoring for health-related behaviour articulates a strong Cartesian distinction, reflected in the very notion of feedback that describes a trajectory from ‘external’ measurements of the body to ‘internal’ reflection. Our general point, as noted by Mol (2009), is that the actual practice of self-monitoring is inescapably more complicated and ‘messy’ than any abstract model, and that presenting a neat enclosed feedback loop inescapably relegates much of the ‘stuff’ of everyday life as extraneous ‘context’. Given the likely promotion of such interventions as means to address a wide range of unhealthy behaviours in the near future, there is consequently a need to draw on a broader, more sociological perspective in discussions of self-monitoring and the use of monitoring devices in order to capture what might be omitted in studies that frame things solely in terms of models of psychological causes and effects (Gately et al., 2007; Ong et al., 2014; Ulucanlar et al., 2013).

## Reconceptualising self-monitoring as an open-ended practice

In the remainder of this article, we examine a trial, called ‘Get Moving’, that tested three forms of self-monitoring to promote physical activity: a paper self-completion physical activity diary; a wrist activity monitor; and a wrist activity monitor with a computer support package, Bluetooth scales, calorie calculator book, low-calorie recipe book and tape measure. In addition to these modes of self-monitoring, all participants (including the control arm) were given a ‘free health check’ both before and after the trial. These consisted of measurement assays^1^ that were not deemed to be part of any intervention, but simply a means to provide baseline and outcome measures.

While the measures in the main study were designed to quantify the effects of the interventions on physical activity, fitness, anthropometry, blood pressure and biochemistry, they did not capture how trial participants actually engaged with or responded to the information they received. Here, we explore what some of the trial participants say they actually did and the extent to which the interventions were adopted and adapted in their everyday lives. One of the concerns we have is that current models of self-monitoring in health research almost invariably draw on a set of abstracted entities – such as ‘the body’, ‘the self’, ‘behaviour’, ‘information’, ‘feedback’ and so on – in order to inform the design of an intervention. In contrast, by shifting from a theoretical-psychological orientation to a more socio-material one, we wish to foreground participants’ own understandings and experiences of using and engaging with the intervention technologies. As a consequence, we are not primarily interested in evaluating what the ‘fit’ was between the intervention as originally theorised and how it was actually used, but rather the ways in which people drew on all or parts of the intervention and incorporated them within their own everyday practices. As a result, by regarding such practices as the source of meaning-making, we emphasise how participation was inevitably constituted by a diverse, and often idiosyncratic, range of social and material factors, placing the very notion of self-monitoring as an inherently psychological process under scrutiny.

Variations of practice theory have been used and described in sociology and anthropology for a number of decades (see, for example, Bourdieu, 1977, 1990; Ortner, 1984; Schatzki, 2001). What, however, is notable is the extent to which it has recently gained prominence in the sociology of health and illness and is often presented as a foil to the widespread adoption of behavioural science within public health. We draw on two themes from this diverse literature to offer an alternative interpretation derived from the participants’ accounts of the Get Moving trial. The first challenge to a psychological model of behaviour, as proponents of Actor–Network Theory argue (e.g. Latour, 2005), is that non-human actors (such as material objects or feedback measurements) should be accorded with as much potential significance as humans. Beyond merely describing them as environmental stimuli that can affect a person, objects, places, medical conditions as well as bodies can all be considered as actors in healthcare practices, with different capabilities and abilities to influence each other (Danholt, 2013). For example, in their discussion of patients’ management of asthma, Danholt and Langstrup (2012) suggest that what is taken to be ‘self-care’ occurs within a broader, more distributed ‘infrastructure’ of persons, things and places. The result is to regard practices as diverse assemblages, comprising specific and local elements that should not be over-generalised or taken out of context.

The second, more specific, argument we adopt by foregrounding practice is a resistance to conceiving the human body as a fixed or stable entity. Mol and Law, for example, have argued that we continually enact our bodies in different ways (Mol and Law, 2004). Rather than a traditional phenomenological account of the body and experience, which relies on a stable distinction between what is ‘internal’ and what is ‘external’, they instead argue that the body is continually being constructed through processes of incorporation and exclusion. Reciprocally, the specific nature and potentials of the body shape the specific ways practices are done. This entanglement between bodies and practices consequently suggests monitoring interventions constitute potentially novel ways to ‘do’ bodies; in other words, they do not record a stable, a priori body, but become part of its ongoing emergence. This arises not simply because the body is known in new ways (e.g. through biomedical measurement) but because the introduction of new practices – in our example, those associated with using the interventions – generates new reactions and responses that shape the body in very material, physical ways. Following Marres’ exploration of the co-articulation of participation and its materialisation (Marres, 2011), this suggests that the specific material ways in which people came to participate is likely to generate a wide range of different and constantly evolving bodies.

Adopting these two orientations serves to resist and reframe some of the assumptions that normally underlie a self-monitoring intervention. Rather than thinking of self-monitoring as an activity of measuring and reflecting on a fixed, objective body which may lead to behaviour as a reasoned response to it, we argue that the potential intervention effect can instead be conceived of as a collection of practices that enact the body in different ways. In addition, by attending to the ways in which participation enlists a wide range of other actors – both other people and other things distributed in space – what is conceived of as singular is made plural. We therefore also show how the inescapable heterogeneity of the manner by which interventions are embedded, or not, in a network of relations means that ultimately each participant engages with their own, unique, intervention. As a consequence, instead of aiming to produce a further refinement of the casual mechanisms by which self-monitoring might be said to ‘work’, we explore an alternative question: To what extent does the notion of a bounded feedback loop fail to account for the many different and creative ways people make sense of, and respond to, a self-monitoring technology? We suggest that the very term ‘self-monitoring’ is not only unhelpful but intrinsically problematic, since it serves to reproduce fundamental ideas and principles that may not be grounded in empirical reality.

## Methods

Following approval from the National Institutes of Health (NHS) Cambridgeshire 2 Research Ethics Committee (Ref. 09/H0308/3), participants were purposefully sampled between February 2012 and October 2013 from the main Get Moving trial to reflect a range of ages and to include both men and women so that diversity of experience could be captured.^2^

Semi-structured narrative-style interviews to allow particular areas of interest to be followed up were undertaken by R.L. either in offices or meeting rooms near participants’ place of work or in dedicated research rooms. Interviews were held once participants had completed the trial and after they had received their health check measurements. We invited people to talk about their participation and the extent to which they thought about their body as the focus of a ‘problem’ or that basis for ‘improvement’. Questions also focused on asking people to describe their trial-related practices in detail. And finally, we asked respondents to describe the material aspects of their participation and how these played a part in the adoption and sometimes adaptation of the intervention received.

The interviews were recorded, sent for external transcription and then compared to the original recordings to ensure accuracy. These verbatim transcripts were imported into NVivo 9 and initially coded using topics derived from the interview guide. As memos and conceptual codes were drawn out from the data, they were discussed, refined and agreed upon with S.C. following his review of the data, and then applied to all transcripts. By doing so, the more obvious descriptive topics were increasingly augmented with cross-cutting analytical themes.

## Findings

In all, 30 trial participants were interviewed, of whom 6 were men and 24 were women, reflecting similar proportions in the main trial. Participant characteristics are shown in Table 1. Somewhat contrary to our expectations, early analysis made it clear that respondents from the different intervention arms often talked about their participation in very similar ways, and that therefore the most pertinent themes were not intervention-specific. It became apparent that findings could most usefully be grouped into two main areas: (1) engagement with the interventions and (2) different modes of knowing.

**Table 1.** Study participant characteristics.

Participant characteristics	*N*
Intervention group	
Diary	10
Activity monitor	10
Activity monitor and support	10
Age (years)	
20–34	10
35–49	6
50–64	14
Sex	
Male	6
Female	24

### Engaging with the interventions

Individuals gave varying reasons for participating in the Get Moving trial. As well as hoping it might encourage them to become more active, others specifically reported that they wanted to use the trial equipment to measure the amount of activity they currently did or compare the data with other forms of self-monitoring equipment they already used (such as smart phone apps or commercial monitoring programmes such as Weight Watchers). Others were motivated by the ‘free health checks’ offered at the end of the study, including a proportion who were seeking a particular measurement, such as a cholesterol test. In addition, many people also expressed more altruistic values, for example, stating that they knew research needed ‘guinea pigs’. Each of these was rarely mentioned on its own; instead, there was often more than one reason why people wanted to participate, suggesting that people from the outset were likely to engage with the equipment in different ways.

What was significant in all the accounts was the extent to which the interventions were experienced as fitting into what participants already did as part of their everyday lives. For a number, whatever trial equipment they had been given, the self-monitoring they were expected to do as a participant simply extended things they already did. These participants described how they were vigilant about aspects of their health anyway and were already consciously aware of many health-related activities, such as what they ate and drank, or how much and what types of physical activity they did. Some even kept records of such things. One, for example, talked about a spreadsheet he had created to work out his fitness and diet, and described how he freeze-dried his food so that it could all be weighed and measured accurately in advance each week:

Yeah, now I have like a full-on like spreadsheet … I’ll have like seven meals a day and then probably there’ll be like macro-nutrient breakdowns … and then how much the make-up of the whole like daily caloric intake and all this stuff. So it’s a super-convoluted spreadsheet now. (Interview 17, 29-year-old male, activity monitor and package intervention, 2 April 2013)

A proportion also monitored additional, non-health, aspects of their lives, indicating that the more general activity of monitoring per se was an established and meaningful practice for them. For example, one participant, who was assigned to the diary intervention group, not only described how she carefully monitored her weight and diet via the Weight Watchers website but also monitored her money using equivalent online resources. As an accountant, she suggested that she was simply the ‘type of person’ who was ‘constantly auditing’ her entire life:

You can probably tell from my finance background, I’m quite an obsessive personality, which is always a good thing for an accountant I think, but it helps you with the WeightWatchers. So I’ve found it relatively easy to stick to their programme, and I’ve lost about two stone 10 pounds from when I started. (Interview 7, diary intervention, 49-year-old female, 18 January 2013)

In contrast, other participants were adamant that they did not normally do any kind of self-monitoring and had little, if any, interest in thinking about health-related activities prior to the trial. For these people, adopting the intervention constituted introducing new and alien tasks into their life. Many of these people stated that electronic devices were physically unattractive or that they looked like electronic police tags for offenders, and that they tried in vain to wear them on different places of their bodies to make them more discreet or comfortable. Others described the technology as ‘awkward’, ‘unsubtle’, ‘uncomfortable’ and so on, suggesting that using the device never really became fully embedded in their routines. There was also variation in the use of the diary. As well as the amount of detail written down, and questioning what was meant to ‘count’ as activity, there was a great deal of variation as to how and when they should be completed. Rather than filled in daily, as they were instructed to do, some completed their diary retrospectively at the end of each week, some randomly left empty pages, while others forgot or abandoned the diary altogether.

The material nature of each intervention impacted not only how people practically managed the task of self-monitoring and the extent to which it was incorporated into everyday practice, but it also affected how they came to make sense of the records produced and what it meant in terms of the status of their body. For many, across all three interventions, the specific form of the information recorded was described as being either unclear or not detailed enough, leaving them to find ways to make sense of it by ‘filling in the blanks’, as one put it. For example, participants described how they regularly tried linking the information back to specific things they remembered doing – and tried to ‘spot’ which measurements might be associated with such things as walking to and from their car, or having gone somewhere for work. But this imperative to make sense of a measurement, to find an explanation for a reading, was not always easy to establish. For example, a 28-year-old female participant using an activity monitor described one occasion when it was not initially clear what had produced an unusual reading of high intensity activity on her graph. She said she had to think long and hard before eventually attributing this to loading wood into her car and subsequently deciding this activity must have been of ‘high impact’ despite not thinking so at the time.

Although only the interventions were framed as the self-monitoring element of the trial, participants also looked at and interpreted the ‘health check’ results they received at the end in a similar way, with participants switching between the two sources of information during interviews in a fluid and interconnected way. For example, the following participant talked through the results from her Actiheart monitor that was used to establish an outcome measure for the trial. It was presented in the form of a graph which showed her level of activity and her heart rate plotted against the date and time:

So I did a lot of dancing at the office outing on that Friday, and I didn’t cycle in that day because we were going out in the evening, I was getting a lift, so you can see the day remains very, very flat. Thursday, you can see I must have gone for a walk at lunchtime, so probably walked up to the Post Office, stopped for a little bit and had a look at the Italian deli probably, and then walked back. (Interview 7, 49-year-old female, diary intervention, 18 January 2013)

These retrospective interpretations of the data was not problematic when the readings gelled with personal experience, but when interpretation was more difficult, people started to question the trustworthiness of the feedback itself. For example, if the person felt that they had been very active and yet little measurement had been recorded, they often doubted the accuracy of the equipment:

… my weight’s the same, my BMI is the same, my waist’s gone down, so how did my body fat go up, you know? That didn’t make sense to me ’cos I think they say most of your fat’s around your stomach, so if your waist’s gone down by two centimetres, how has your body fat gone up by two percent? (Interview 25, 42-year-old female, diary intervention, 14 May 2013)

Participants also queried how much movement the wrist monitors could actually pick up and suggested that activities such as cycling and skiing might be under-recorded because their arms remained largely stationary.

Given this variation in the engagement and use of the trial equipment across the three subgroups, the 30 interviews could be said to describe 30 very different interventions, and hence 30 ways in which the monitoring practices enacted a body. Each person had a story to tell and a description of how they did not simply respond to the task of self-monitoring passively, but actively found ways to incorporate it, adjust it or occasionally exclude it altogether. Similarly, people variously described ways in which the equipment, or the protocols relating to their use, was resisted, altered or refigured in relation to their own expectations and understandings. Summarising this as a process of possible appropriation and adaptation rather than adoption of an inflexible intervention (Storni, 2010) not only means that the intervention can never be said to be stable or standardised but suggests that monitoring itself is likely to have had many different meanings, potential effects and forms of productivity.

### Modes of knowing

In addition to the degree to which the intervention was felt to extend existing practices or was experienced as an alien imposition was a general theme questioning what kind of knowledge the monitoring produced.

One general frustration in relation to this was the fact that the monitoring was intended to be done individually, with no support from others. For example, a 58-year-old female participant responded that she was disappointed that the feedback from her activity monitor did not make her reflect on her activity and encourage her to be more active, as she expected it to. She described how she consequently stopped because it felt more like being watched anonymously than receiving support or encouragement from another person who could look at the data with her:

I thought that there was somebody … who was going to look at my graphs and do kind of things with it [the data from the graphs] and initially that’s why I got it [the intervention] and it didn’t make any impact because after two weeks I thought ‘Oh God, yeah, I don’t want to do it’ [laughs] … I need to have that other person and it has to be a person who is kind of there encouraging me and pushing me and thinking, you know, you need to do this [increase physical activity] … (Interview 6, 58-year-old female, activity monitor only intervention, 4 January 2013)

In their everyday adoption and engagement, the different kinds of feedback information were often shown to, and discussed with, friends and family members. And because the trial was located within an occupational setting, a number of participants also discussed and compared their results with work colleagues – sometimes with others who were also undertaking the trial. Involving someone else was often a means to share and enlist support. But more than this, other people were also very active in the monitoring process; participants commonly described how friends or relatives commented on, helped interpret and even gave advice in response to the readings. They also not only accompanied participants in any physical activity, for example, going for walks or jogging, but also started monitoring their own activity levels. For example, during one interview, a participant described how both her husband and her daughter became increasingly involved:

… Although I was the one writing in the book [the diary] it, they’d say ‘oh yeah, you know, if we do this you can put it in your book’ and ‘oh yes, yes’, you know. But it was as much, you know, it spurred them as well, definitely. Definitely. (Interview 19, 50-year-old female, diary intervention, 15 April 2013)

Such involvements served as a means of not only offering support but also establishing the monitoring as an inherently social activity, and consequently meaningful in a relational way. As a result, the knowledge produced by the monitoring was often indirectly referred to as an account of this social activity, rather than a record of the status of the individual body.

Alongside this means of appropriation through extending the intervention within social networks, participants frequently mentioned how they drew on their own markers of health and physical activity to either complement or contest the trial information. For example, they commonly assessed their level of physical activity using the fit of their clothing, such as which notch on a belt was used, or noting when clothes had become too tight or they had to buy a larger size:

… my clothes were feeling tight, they wouldn’t fit, then I was having to buy bigger clothes. I think some of my clothes that I did have stretched out a bit, and when I had to buy a pair of replacement trousers when I fell over and I had to buy a size 16, I think that was the real turning point, yeah. (Interview 7, 49-year-old female, diary intervention, 18 January 2013)

Others talked about how they could assess changes to their body shape in the mirror or judge their fitness according to how they felt after physical exertion, such as climbing the stairs or running for a bus. Many interviewees went on to use these personal evaluations as the basis for comparing themselves with others, such as their work colleagues or friends and family members. Often people would try to take into account age, gender and lifestyle as well as the social context that determined how they related to others in order to achieve some kind of meaningful comparison. For example, one participant described how he drew on a range of elements he thought were particularly relevant:

… if I’m playing football, I kind of know such-and-such person does this for a living and he’s still able to run as fast as me at the age of 35 or 40, you know, and then I go like, ‘OK, this is just not good on my part’ So that is … yeah, so that’s a big incentive. (Interview 15, activity monitor only intervention, 29-year-old male, 13 March 2013)

The diverse ways in which people assessed their own bodies and health status represent methods felt to be useful and meaningful, not because they promised accuracy or objectivity but because they were techniques that drew on, and co-opted, aspects of everyday life. In contrast, the self-monitoring practices associated with the trial generated information that was often fundamentally disassociated from people’s normal activities, and hence was frequently not considered intrinsically meaningful.

Ostensibly, these more embodied, experiential modes of knowing potentially competed with the more abstract self-monitoring measurements and the ways in which it demarcates the individual from their environment. However, the relationship between participants and the interventions was often more complex, and akin to an ‘ontological choreography’ (Cussins, 1996), by which people and the monitoring equipment often became entangled to produce diverse experiences of agency and objectification held in tension with one another. The effect was that the intervention equipment was not necessarily talked about as being ‘outside’ or beyond the body, providing measurements from a distance, but rather that in the process of its appropriation, it could lead to a modified sense of the body through the very act of monitoring. This suggests that ultimately participation was not defined by the recording or flow of information in a loop to precipitate behavioural change, but rather by the very material and embodied acts of simply *doing* the monitoring and the ways in which this led to new practices and ways of knowing the body. Thus, while the study might ostensibly present itself as one that tested ‘self-monitoring’, in practice, the intervention was not solely about ‘self’ nor about just ‘monitoring’. Both the emphasis on ‘the self’ and that a singular mode of monitoring was being evaluated fails to acknowledge the multiple and creative ways in which participants adopted the task they were asked to do, and how they invariably drew on a range of different modes of knowing rather than just that provided by the intervention technologies.

## Discussion

A retrospective interview study such as this is clearly limited by the fact that the interview itself is likely to shape the way things were recalled. Nevertheless, respondents gave very rich and detailed accounts, drawing on many aspects of their own experience and giving meticulous descriptions about the extent to which the intervention they received was embedded, or not, in their everyday lives. From the interviews, it is clear that frequently there were many components that became involved, or enlisted, as the interventions were engaged with and that as a result the influence of the intervention was often complex, multiple and changeable.

One of the most apparent things we describe was that in cases where people were already engaged in some kind of measurement activity and reflection, the intervention they received tended to be incorporated with relative ease. However, for people whose lives contained little that resembled the logic of self-monitoring, the intervention was far more difficult to adopt; indeed, sometimes it was actively resisted. This distinction was not, however, associated with a particular intervention, the type of equipment or the nature of self-monitoring or any overt demographic characteristic of the participant.

It is also clear that the process of self-monitoring nearly always involved more than the individual participant. Friends, colleagues and family members invariably were enlisted in a variety of different ways. They often viewed, interpreted and talked about the results; they sometimes supported participants to use the equipment; and they encouraged participants to increase their physical activity, to the extent that some were happy to do the various activities as well. Interviewees also incorporated people indirectly into their ways to make monitoring practices meaningful, by comparing themselves with others. Such comparisons were not made in relation to abstract averages, population-based means or clinical thresholds, but were much more personalised, as people drew on existing ideas of similarity – for example, by estimating the age of a stranger or how heavy a colleague might be – in order to then establish if here were then any relevant areas of difference. While much behaviour change literature does acknowledge the role of social networks and support (Kelly et al., 1991), this is generally absent in accounts of self-monitoring because the phrase itself brackets off such relational aspects as not being intrinsic to what ‘self-monitoring’ fundamentally consists of. In contrast, our study shows that from the participants’ own perspectives, this bracketing off is not only unhelpful but actually has the effect of excluding many of the ways in which an intervention might be said to engage with people and their bodies.

Drawing on De Certeau’s (1984) general arguments about the everyday practices of users and their adoption of various tactics, the ways participants creatively negotiated the interventions within a broad and distributed social and material ‘art of practice’ might, from the perspective of the trialists, be viewed as a form of resistance to the principles of monitoring and feedback. However, rather than seeking ‘shortcuts’, we have presented how many people described tactics that ostensibly did the opposite; engaging with the interventions often led people to actively enrol more elements, not less, transforming what were intended to be a discrete and individualised intervention into more complex sets of relationships and practices. This process of expanding and ‘opening up’ the interventions in order for them to potentially become embedded in everyday life suggests that what is nominally described as self-monitoring in practice refers to the incorporation of technology and measurement within a range of other forms of knowledge, activities and social networks. The result is not a reasoned, contained or calculable process, but rather a much more open one of bricolage that draws on many sources to extend into, and potentially enlist, diverse components. Accounts reveal how everyday practices and processes of meaning-making often generated long and convoluted journeys that included other people and other objects, each of which is likely to contribute to how interpretations are made.

We also discussed at the beginning how, in everyday life, people gain a sense of their body not simply through conscious reflection but ways of knowing gained through doing. These embodied practices produce a sense of the body through less conscious, rational assessments. While for some participants mention of these sometimes resembled quite phenomenological accounts, other ways of knowing the body arose from their engagements with other people and things beyond the traditional sense of the body. In other words, the more extended descriptions of participating in monitoring activities in parallel gave rise to more dispersed ways of knowing the body. This recursive relationship between the experience of knowing and the experience of the body in practice clearly contrasts with the model of a feedback loop, in which knowledge about the body and the body itself are distinct entities.

In this article, we have not looked in depth at how specific elements link together and achieve this for individual participants. Nevertheless, we have illustrated how an approach which focuses on everyday practices, understandings and bodies as being contingent on action suggests that the constructs underpinning self-monitoring and feedback are not only limiting but often at odds with accounts made by people themselves. Descriptions of the complexities involved in gaining, making sense of and then acting upon information illustrate the extent to which an exchange of knowledge is not necessarily straightforward or restricted, and that the process frequently incorporates more components than medical researchers tend to assume. Furthermore, our findings suggest that, in fact, often what is significant is not the specific information that might travel or be translated, but the very sense of connecting and constructing. Given the renewed interest more generally in care as a relational quality, rather than something bestowed by one person on to another, this perhaps implies at least one dimension of any successful intervention must be the extent to which it can help foster meaningful and productive relations of everyday practice.

Finally, since participants engaged with the monitoring activities so differently, frequently combining diverse types of knowledge and welcoming the active contributions of other people, it may be time to consider jettisoning the term ‘self-monitoring’ altogether: As we have argued, our study problematises both the idea of the self and of monitoring. This has implications for the development of health interventions in the future, not crudely in relation to who might use and benefit from monitoring equipment, but the extent to which the notion of an enclosed causal pathway can ever capture the complex and often multiple ways in which people generate and engage with information and practices relating to their bodies.

## Notes

1. The ‘health check’ consisted of clinical tests (total body weight, body mass index (BMI), total body fat, blood pressure, HbA1C, total cholesterol) and measures of fitness and physical activity (time spent on treadmill test, VO2 max, time spent at moderate-to-vigorous physical activity and graphs of activity and heart rate from the Actiheart monitor worn for 6 days during the trial).

2. This recruited participants aged 18–65 years who worked on the Cambridge Biomedical Campus; potential participants were excluded if they were physically active (scoring ≥30 on the Godin Leisure-Time Exercise Questionnaire for moderate-to-vigorous activities, Godin and Shephard, 1985), had been instructed by their general practitioner (GP) not to undertake regular physical activity, were pregnant or unable to walk briskly for 15 minutes without help, or if they were participating in another clinical trial (see Cooper et al., 2015 for further details of the trial).
